# Identifying targets for increased biogas production through chemical and organic matter characterization of digestate from full-scale biogas plants: what remains and why?

**DOI:** 10.1186/s13068-022-02103-3

**Published:** 2022-02-10

**Authors:** Eva-Maria Ekstrand, Annika Björn, Anna Karlsson, Anna Schnürer, Linda Kanders, Sepehr Shakeri Yekta, Martin Karlsson, Jan Moestedt

**Affiliations:** 1 Department of Thematic Studies – Environmental Change, Linköping University, 58183 Linköping, Sweden; 2 Biogas Research Center, Linköping University, 58183 Linköping, Sweden; 3 Scandinavian Biogas Fuels AB, Holländargatan 21A, 111 60 Stockholm, Sweden; 4 Department of Molecular Sciences, Swedish University of Agricultural Sciences, Uppsala BioCenter, Box 7025, 750 07 Uppsala, Sweden; 5 Purac AB, Box 1146, 221 05 Lund, Sweden; 6 Molecular Biotechnology, Department of Physics, Chemistry and Biology, Linköping University, 581 83 Linköping, Sweden; 7 Department of Biogas R&D, Tekniska verken i Linköping AB, Box 1500, 581 15 Linköping, Sweden

**Keywords:** Anaerobic digestion, Degradation efficiency, Residual methane potential, Macromolecules, Full-scale biogas plants, Trace metals, Ammonia, Enzyme activity, Biogas, Digestate

## Abstract

**Background:**

This study examines the destiny of macromolecules in different full-scale biogas processes. From previous studies it is clear that the residual organic matter in outgoing digestates can have significant biogas potential, but the factors dictating the size and composition of this residual fraction and how they correlate with the residual methane potential (RMP) are not fully understood. The aim of this study was to generate additional knowledge of the composition of residual digestate fractions and to understand how they correlate with various operational and chemical parameters. The organic composition of both the substrates and digestates from nine biogas plants operating on food waste, sewage sludge, or agricultural waste was characterized and the residual organic fractions were linked to substrate type, trace metal content, ammonia concentration, operational parameters, RMP, and enzyme activity.

**Results:**

Carbohydrates represented the largest fraction of the total VS (32–68%) in most substrates. However, in the digestates protein was instead the most abundant residual macromolecule in almost all plants (3–21 g/kg). The degradation efficiency of proteins generally lower (28–79%) compared to carbohydrates (67–94%) and fats (86–91%). High residual protein content was coupled to recalcitrant protein fractions and microbial biomass, either from the substrate or formed in the degradation process. Co-digesting sewage sludge with fat increased the protein degradation efficiency with 18%, possibly through a priming mechanism where addition of easily degradable substrates also triggers the degradation of more complex fractions. In this study, high residual methane production (> 140 L CH_4_/kg VS) was firstly coupled to operation at unstable process conditions caused mainly by ammonia inhibition (0.74 mg NH_3_-N/kg) and/or trace element deficiency and, secondly, to short hydraulic retention time (HRT) (55 days) relative to the slow digestion of agricultural waste and manure.

**Conclusions:**

Operation at unstable conditions was one reason for the high residual macromolecule content and high RMP. The outgoing protein content was relatively high in all digesters and improving the degradation of proteins represents one important way to increase the VS reduction and methane production in biogas plants. Post-treatment or post-digestion of digestates, targeting microbial biomass or recalcitrant protein fractions, is a potential way to achieve increased protein degradation.

**Supplementary Information:**

The online version contains supplementary material available at 10.1186/s13068-022-02103-3.

## Introduction

Anaerobic digestion (AD) is a well-established technology for both generating renewable biogas and valorizing organic waste fractions [1]. Biogas consists primarily of methane and carbon dioxide, with methane being a versatile energy carrier that can replace fossil fuel in vehicles, heat and power production, and industrial processes [1]. In addition to biogas, AD produces a nutrient-rich residue (i.e. digestate) that can be used as fertilizer in agriculture, thereby recycling nutrients between urban and rural areas [2]. Due to the multifunctionality of the process and its high-value outputs, AD can be seen as central to achieving a circular bioeconomy. Consequently, the number of AD plants in Europe has increased in recent years, and waste and residues from agriculture and industry as well as municipal organic waste and sewage sludge are now increasingly treated in biogas plants [3]. To reach environmental goals and to optimize economic output, AD plants should be operated at high efficiency, i.e. at a high biogas yield per reactor volume and time combined with a high degree of degradation. The degradation efficiency of the biogas process is important for both biogas production and nutrient levels in the digestate and is also decisive for the digestate’s residual methane production [4]. A well-digested material with low residual methane potential (RMP) will decrease greenhouse gas (GHG) emissions associated with subsequent digestate storage.

The efficiency of a biogas process depends on several factors, often interlinked, including the composition and pre-treatment of the ingoing substrate, operational parameters such as organic load, hydraulic retention time, mixing and digester fluid behaviour and temperature, as well as digester technology [5]. In addition, an active and well-synchronized microbial community is needed [6]. AD processes proceed through several degradation steps performed by metabolically linked microbial groups, typically operating in a synchronized manner [5, 7]. In the first step, hydrolysis, extracellular enzymes (e.g., lipases, proteases, and cellulases) attack and break up fat, proteins, and carbohydrates. The rate of this step is strongly dependent on the substrate accessibility, which in turn depends on the chemical composition [8]. In the next steps, the hydrolysis products (i.e. oligo- and monomers) are converted to fatty acids (via acidogenesis) and alcohols, followed by further oxidation of the acids mainly to acetic acid and hydrogen/carbon dioxide (via acetogenesis). In the final step, acetate and H_2_/CO_2_ are converted to methane by acetotrophic and hydrogenotrophic methanogens, respectively. The composition and activity of the prevailing microbial community depend on both the external operational parameters and internal environmental conditions of the digester (e.g., pH, ammonia levels, and volatile fatty acid concentration [6]). In addition, the levels and availability of various ions and trace metals are crucial for microbial activity (mainly at the enzyme level) and thus for substrate degradation efficiency [9]. Moreover, the proportion of acetotrophic versus hydrogenotrophic methanogenesis depends strongly on the prevailing environmental conditions. At a high ammonia level or a thermophilic temperature, methane formation proceeds mainly via syntrophic acetate oxidation (SAO) coupled to hydrogenotrophic methanogenesis [10].

The AD of substrates and the subsequent methane production can be improved by various operational strategies such as: (a) pre-treatments to break up complex macromolecular structures for increased substrate accessibility and microbial degradation (reviewed by Atelge et al. [11] and Mirmohamadsadeghi et al.[12]); b) co-digestion approaches or the use of process additives, often trace metals, to improve the nutrient balance of the AD process (reviewed by Mata-Alvarez et al. [13]); c) adjusting the organic loading rate (OLR) and hydraulic retention time (HRT) or implementing post-digestion, i.e. implementing a second step of digestion to ensure sufficient degradation time; or d) using thermophilic operational conditions for enhanced degradation rates [14]. The optimal strategy to use will vary depending on the substrate mix.

Based on RMP values determined in different studies, it is clear that the residual organic matter in digestates varies and can have significant biogas potential [4, 15, 16]. However, knowledge of the factors dictating the size and composition of this residual fraction and of how they correlate with RMP is currently lacking. The aim of this study was therefore to generate additional knowledge of the compositions of the residual fractions in digestates and to understand how they correlate to various operational and chemical parameters, in order to formulate strategies for enhanced methane production. This was undertaken by characterizing the composition of both the substrates and digestates from nine full-scale biogas plants, including both the main digesters and post-digesters, when present. The residual organic fractions were then linked to parameters such as substrate type, pH, levels of trace metals and ammonia, viscosity, operational parameters (e.g., OLR, HRT, and temperature), RMP, and enzyme activity (i.e. cellulase, protease, and lipase). The selected plants operated with different categories of substrates: four plants co-digested primarily food waste (FW), two plants operated on plant-based agricultural wastes (AW), one plant operated on AW together with manure (AWM), and two plants were wastewater treatment plants (WWTPs) using a mix of primary and waste activated sludge.

## Results

### General process conditions

In total, nine plants were sampled in this survey, eight wet digestion plants and one high solids digestion plant. All plants operated in continuous mode, seven of the AD plants were operating under stable conditions with low volatile fatty acids (VFAs) concentrations and pH levels above 7.2 (Table 1). However, analyses of digestates from two of the plants, FW2 and FW-TD, showed elevated levels of total VFAs (1360 and 8450 mg/L, respectively), yet at maintained high pH (> 7.8). In FW2, the primary VFA was acetate, whereas propionate in FW-TD represented a high fraction of the total VFAs, e.g., 7300 mg/L. The total solids (TS) were highest in the plants treating agricultural substrates (AW and AWM) and lowest in the WWTPs. All plants operating with a post-digester, i.e. a second digestion step degrading the outgoing material from the first digestion step, displayed decreased TS in the post-digester relative to the main digester (Table 1). The volatile solids (VS) reduction of the processes was 48–77%, with highest VS reduction in FW1, FW3, AW1, and AW2 at 77–78% and lowest VS reduction in FW2 at 48% (Table 1). The ammonium and free ammonia levels varied among the plants, i.e. 0.8–3.8 g/kg for NH_4_^+^-N and 0.02–0.74 g/kg for NH_3_-N. FW1, FW2, and FW-TD all had NH_4_^+^-N above 3.4 g/kg, while the NH_3_ content in FW-TD was twice as high (0.74 g/kg) than in FW1 and FW2 (Table 1).

**Table 1:** Process performance parameters of digesters and post-digesters at full-scale biogas plants at the time of sampling

Plant	Sample point	TS (%)	VS (% of TS)	VS red. (%)	pH	NH_4_^+^-N (g/kg)	NH_3_-N (g/kg)	VFA (mg/L)
FW1	D	5.3	73.6	70	7.8	3.4	0.34	80
	PD	4.3	71.5	77	7.8	3.6	0.35	40
FW2	D	3.9	66.7	48	7.9	3.6	0.31	1360
	PD	3.8	65.5	50	7.9	3.8	0.35	190
FW3	D	4.1	69.6	75	7.5	2.4	0.10	40
	PD	3.8	67.9	77	7.7	2.5	0.13	20
FW-TD	D	11.6	66.6	65	7.8	3.8	0.74	8450
AW1	D	10	88.2	67	7.6	2.4	0.12	150
	PD	7.2	86.0	78	7.5	2.3	0.08	60
AW2	D	8.6	81.8	66	7.3	3.3	0.09	320
	PD	6.3	77.3	77	7.8	2.4	0.20	2
AWM-A AWM-B	D	7.3	74.1	62	7.9	2.0	0.18	370
	D	7.4	74.2	62	7.8	3.4	0.26	420
WWTP1	D	3.2	66.5	55	7.9	1.4	0.15	20
WWTP2-A WWTP2-B	D	1.6	64.5	63	7.2	0.8	0.02	20
	D	1.7	65.7	61	7.4	0.9	0.02	40

*FW* food waste, *TD* thermophilic dry digestion, *AW* plant-based agricultural waste, *AWM* agricultural waste + manure, *WWTP* wastewater treatment plant, *TS* total solids, *VS* volatile solids, *VS red* VS reduction, *VFA* total concentration of volatile fatty acids, *D* main digester, *PD* post-digester

### Cluster analysis

**Fig. 1:**
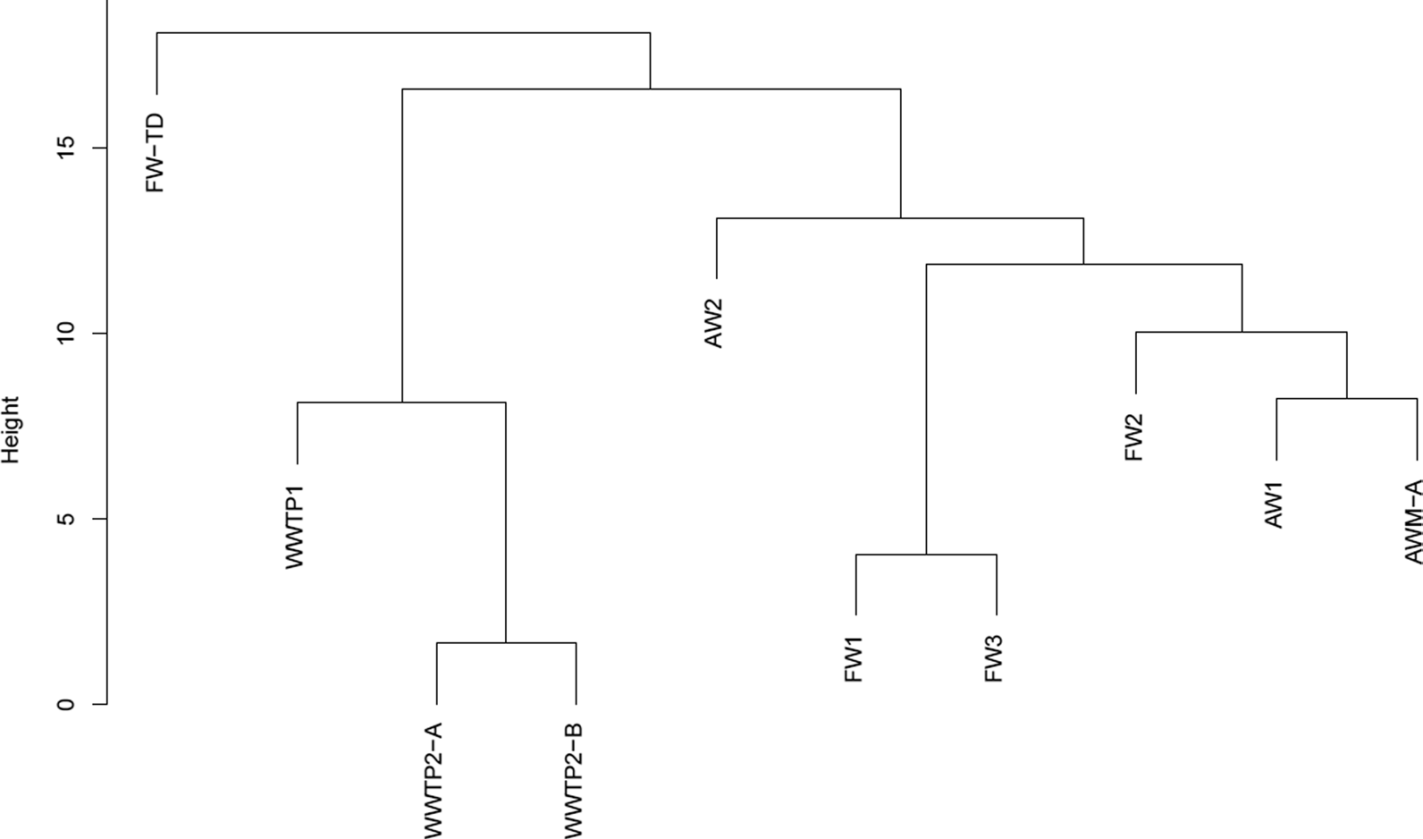
Hierarchical cluster dendrogram of all complete datasets; FW = food waste, TD = thermophilic dry digestion, AW = plant-based agricultural waste, AWM = agricultural waste + manure, and WWTP = wastewater treatment plant

Cluster analysis of the operational and chemical parameters for the plant digestates (see Table 2, Table 1, and Additional file 1) indicated the clear separation of the sewage sludge digesters from digesters using other substrate types (Fig. 1). The food waste processes, FW1 and FW3, were grouped together; FW2 instead clustered with the AW-based processes AW1 and AWM, whereas the digestate from FW-TD (TD = thermophilic dry digestion) was found to differ greatly from all other digesters (Fig. 1).

**Table 2:** Substrate composition and operational parameters of the full-scale processes included in the study

Plant	Substrate composition (% of ingoing volume)	OLR (ton VS/m^3^·day)	HRT (days)	T (°C)	Sampling points
FW1	Food waste (60%), slaughterhouse waste (25%), industrial waste (15%)				Substrate mixture
		4.2	35	42	Digester
			20	41	Post-digester
FW2	Food waste, vegetables, moulded food packages, slaughterhouse waste, food industry waste, pig manure, grease separator sludge^1^				Substrate mixture
		3.0	25–35	37	Digester
			5	37	Post-digester
FW3	Organic fraction of municipal household waste (100%)				Substrate mixture
		3.8	27	38	Digester
			7	36	Post-digester
FW-TD	Organic fraction of municipal household waste (98%), garden waste (2%)				Substrate mixture
		4.7	27	55	Digester
AW1	Starch slurry (80%), cereals (18%), forage and corn silage (2%)				Starch slurry
					Cereals
					Corn silage
		5.8	55	38	Digester
			40	38	Post-digester
AW2	Crop silage (46%), corn silage (26%), sugar beets (16%), cereals (12%)				Substrate mixture
		3.1	40	38	Digester
			45	38	Post-digester
AWM	Pig manure (52%), grease sludge (16%), cow manure (12%), agricultural residues (12%), chicken manure (3%), deep litter (3%), slaughterhouse waste (2%)				Manure slurry + grease
					Remaining fractions (see left)
		3.4	55	38	Digester A
		3.4	55	38	Digester B
WWTP1	Primary and activated sewage sludge (98%), grease separator sludge (2%)				Substrate mixture
		2.5	20	38	Digester
WWTP2-A WWTP2-B	Different substrate fractions for digester A and B, see below:				Primary sludge
					Activated sewage sludge
					Grease separator sludge
	Primary (80%) and activated (20%) sewage sludge	1.5	17	37	Digester A
	Primary (80%) and activated (12%) sewage sludge, grease separator sludge (8%)	1.8	15	37	Digester B

^1^ Volume fractions of the substrate were not provided by the biogas plant

Several plants received a combination of substrates and were designated as FW/AW/WWTP relative to the main substrate fraction. *FW* food waste, *FW-TD* thermophilic dry digestion of food waste, *AW* plant-based agricultural waste, *AWM* agricultural waste + manure, *WWTP* wastewater treatment plant, *OLR* organic loading rate (ton VS/m^3^ day), *HRT* hydraulic retention time (days), *T* temperature (°C)

### Organic composition and degradation efficiencies

**Fig. 2:**
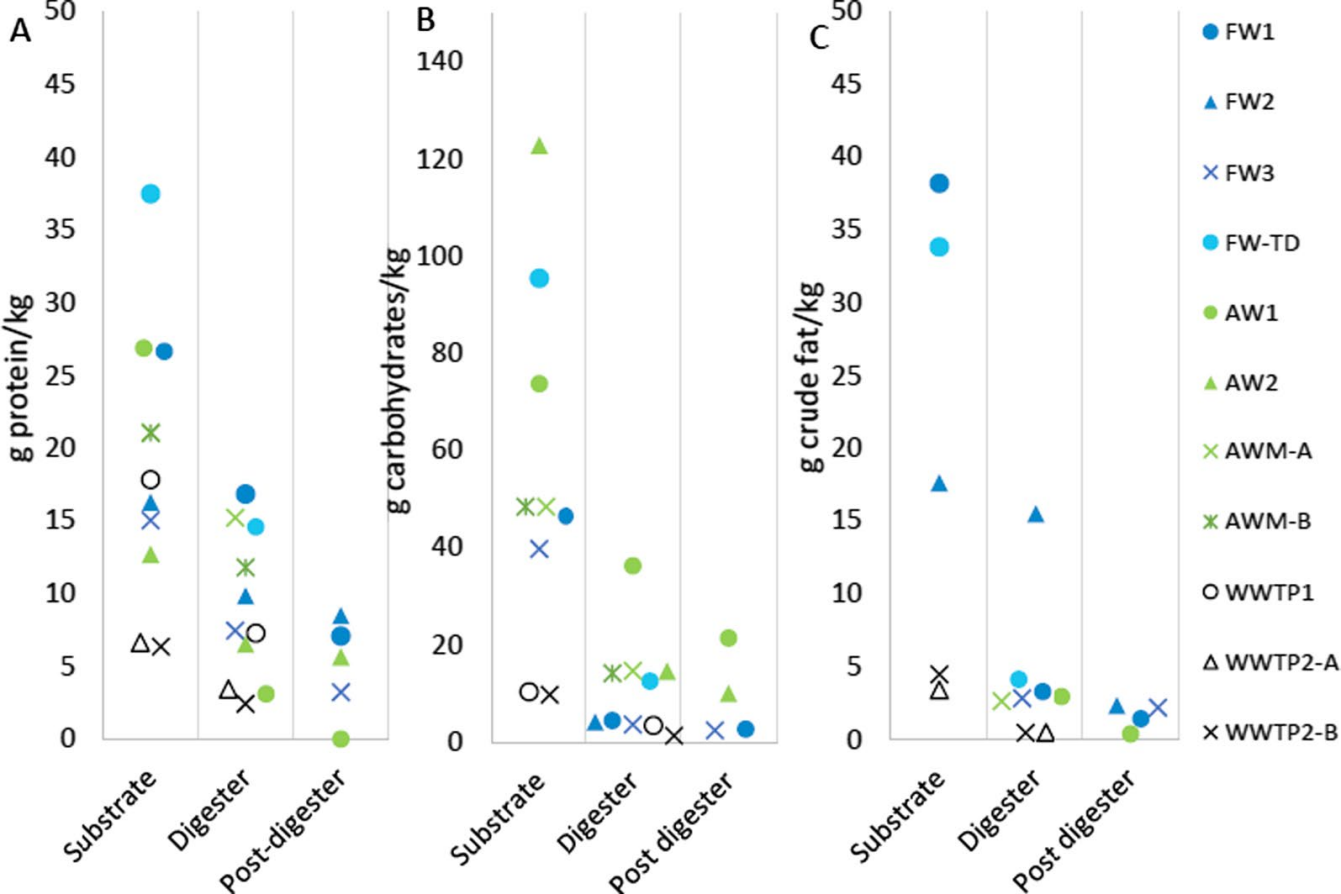
The amounts of **A** proteins, **B** carbohydrates, and **C** crude fat per kg ww in the different steps of the full-scale biogas plants (i.e. substrate, digester, and post-digester); FW = food waste, TD = thermophilic dry digestion, AW = plant-based agricultural waste, AWM = agricultural waste + manure, WWTP = wastewater treatment plant

The analyses of protein, carbohydrate, and fat contents in the substrates and different digestion steps revealed both differences and general trends among the investigated plants (Fig. 2). The higher TS content in the substrate mixtures of AW1, AW2, and FW-TD resulted in higher absolute concentrations of the macromolecules than in substrates of WWTPs. Carbohydrates represented the largest fraction of the total VS (32–68%) in all substrates, except in WWTP1 and FW2, where protein was the dominant fraction.

**Fig. 3:**
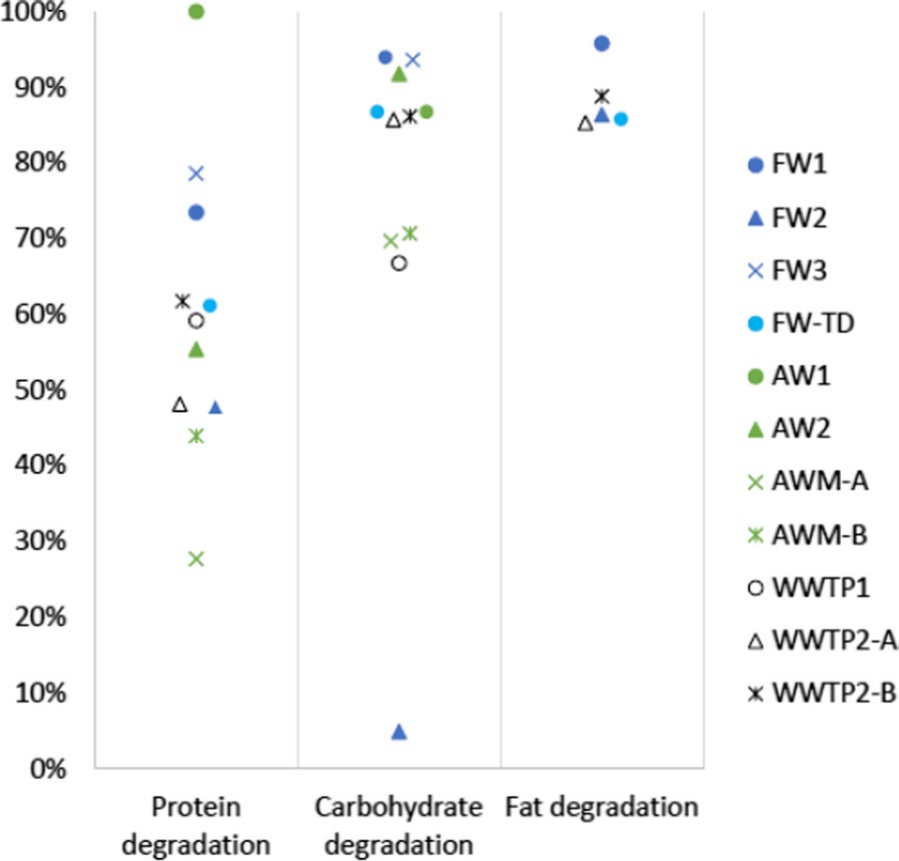
The overall degradation efficiencies (%) for protein, carbohydrates, and fat in the full-scale biogas plants; FW = food waste, TD = thermophilic dry digestion, AW = plant-based agricultural waste, AWM = agricultural waste + manure, WWTP = wastewater treatment plant

In the plants treating FW, protein was the most abundant residual macromolecule in the digestate of the main digesters (11–21 g/kg wet weight [ww]), while carbohydrates were generally at lower levels (3.7–4.5 g/kg). However, within the FW group, the fat levels were significantly higher in FW2 at 16 g/kg, than in FW1 and FW3, at 3.6 and 3.1 g/kg, respectively. In the FW plants with post-digesters (applying post-digestion), the protein content was further reduced to 7–12 g/kg (Fig. 2A), resulting in an overall protein degradation efficiency of 48–79%, with the lowest degradation efficiency in FW2 and the highest in FW3 (Fig. 3). Similarly, post-digestion in the FW plants reduced the carbohydrate and fat contents to 2.5–4.2 g/kg and 1.5–2.2 g/kg, respectively, reaching overall degradation efficiencies of 87–94% and 86–96%, respectively. An exception to this was FW2, which reached a sugar degradation efficiency of only 5%, likely due to a very low intrinsic sugar content in the substrate (Fig. 3; Additional file 1). A pattern similar to that in FW was observed in the WWTP, with protein representing the most abundant macromolecule (3.3–8.8 g/kg), exceeding both carbohydrates (1.4–3.5 g/kg) and fat (0.5 g/kg) (Fig. 2). The WWTPs, neither of which applied post-digestion, reached overall degradation efficiencies of 48–62% for protein, 86% for carbohydrates, and 85–89% for fat (Fig. 3).

In the main AW digesters, carbohydrates were the most abundant macromolecule, found in concentrations of 13–36 g/kg, while protein concentrations were lower at 11–21 g/kg and fat, as in the other plant types, was almost completely degraded (i.e. 2.9–3.5 g/kg; Fig. 2). After post-digestion, both carbohydrates and proteins were further degraded and reached 10–22 g/kg and 8–13 g/kg, respectively. The high carbohydrate content reflected the high carbohydrate concentration in the substrate (50–124 g/kg) rather than resulting from poor degradation, as the carbohydrates were degraded rather efficiently (71–92%; Fig. 3). The protein degradation efficiencies (excluding estimated protein formed through biomass growth during degradation, Y_prot_) varied considerably among these plants, ranging from 27–44% in AWM to about 55% and 100% in AW2 and AW1, respectively (Fig. 3).

Notably, and common in all plants except AW1 and WWTP1, was that the fraction of VS comprising proteins increased from substrate to main digester.

### Residual methane potential

**Fig. 4:**
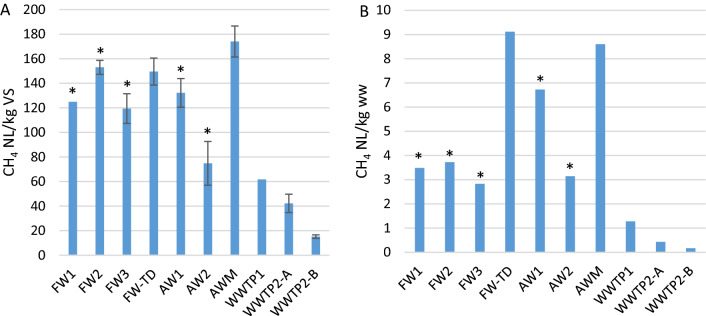
Residual methane potential in digestates from the last AD step, expressed in both A) NL/kg VS and B) NL/kg; FW = food waste, TD = thermophilic dry digestion, AW = plant-based agricultural waste, AWM = agricultural waste + manure, WWTP = wastewater treatment plant (A and B are two digesters at the same plant, see Table 2), N = normalized to 1 atm pressure and 273 K; plants marked with an asterisk (*) had post-digesters

The RMP varied considerably among the studied plants, being 15–170 NL/kg VS and 0.2–9.1 NL CH_4_/kg ww (Fig. 4). The specific RMP (NL/kg VS) was lower in the WWTPs and AW2 than in AW1, AWM, and the FW digesters, indicating that the residual VS degradability (in terms of gas potential) was relatively low in these plants. Among the AW digesters, AW2 had a notably low RMP, both per g VS (75 NL/kg VS vs. 130–170 NL/kg VS) and per kg ww (3.1 NL/kg vs. 6.7–8.6 NL/kg).

The volumetric RMP (NL/kg) was largely dependent on the outgoing TS content, so the three reactors with the highest TS (i.e. FW-TD, AW1, and AWM) also had the highest RMP (L/kg ww) (Fig. 4).

### Macromolecule degradation

For three of the plants (i.e. FW1, FW-TD, and WWTP2), a mass balance was calculated for the organic macromolecules, from substrate to digester and post-digester, including protein, protein from biomass, cellulose, hemicellulose, lignin-like structures, free sugars, and crude fat.

**Fig. 5:**
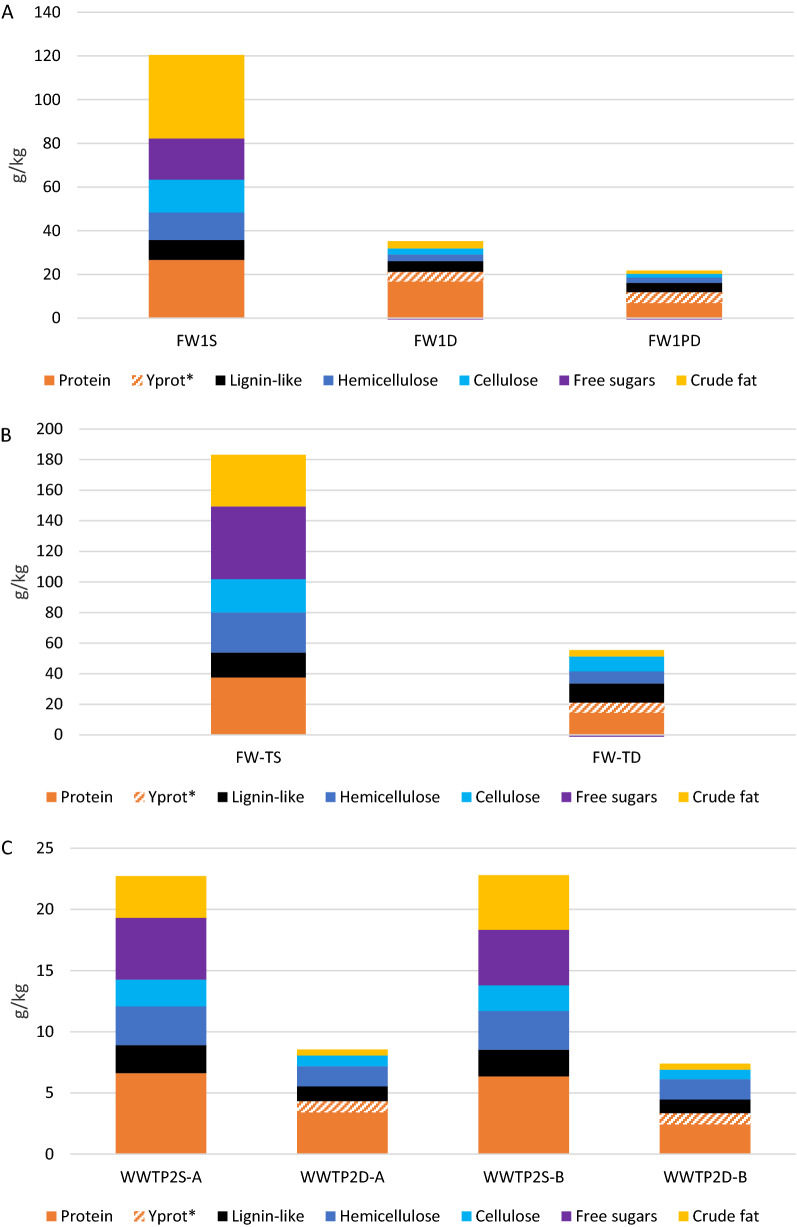
Concentrations of macromolecules in **A** FW1, **B** FW-TD, and **C** WWTP2-A and B with the letters S, D, and PD signifying substrate, digester, and post-digester, respectively; Y_prot_ represents new protein formed due to VS degradation and biomass growth

The substrate added to FW1 had a total VS content of about 120 g/kg ww (12% of total ww). The substrate roughly comprised 22% proteins, 8% lignin-like structures, 39% carbohydrates (including 10% hemicellulose, 12% cellulose, and 16% free sugars), and 32% crude fat (Fig. 5A). However, after digestion (35 days average HRT, main digester), the degradation of free sugars (100%), hemicellulose (76%), cellulose (82%), and fat (91%) resulted in low outgoing concentrations of these macromolecules to the post-digester. The major fraction left was proteins, which had only decreased from 27 to 17 g/kg (a 37% reduction, excluding Y_prot_). During post-digestion, the residual protein was further degraded, yet 7 g/kg remained, resulting in overall protein degradation of 74% (Fig. 5A). Considering the protein formed during biomass growth (Y_prot_), the total residual protein in the post-digestate increased to 12 g/kg, representing 55% of the total VS, and the overall reduction of proteins ended at 55%. Residual carbohydrates (i.e. cellulose + hemicellulose) were below 4.0 g/kg and residual lignin-like structures were 4.3 g/kg.

In FW-TD, the only dry-digestion plant in the study, the ingoing VS content was high at 180 g/kg (18%; Fig. 5B). As in FW1, carbohydrates represented the major substrate fraction (52%), although the substrate of FW-TD contained a relatively larger fraction of hemicellulose (15% VS vs. 10% VS in FW1). Moreover, compared with FW1, the fat content in the substrate was lower in FW-TD (11%), likely due to the rather large fraction of fat-rich slaughterhouse waste relative to FW1 (Table 2). Proteins in the substrate in FW-TD were 20% VS, only slightly lower than in FW1. Free sugars and crude fat were efficiently degraded (100% and 88%, respectively), while proteins, cellulose, and hemicellulose were relatively more resistant to degradation, reaching degradation efficiencies of 44, 55, and 70%, respectively. As in FW1, protein (14.6 g/kg) together with Y_prot_ (6.5 g/kg) represented the major residual organic fraction in the FW-TD digestate (42%), whereas cellulose and hemicellulose remained to a larger extent (i.e. 9.7 g/kg and 7.9 g/kg, respectively) versus in FW1.

In WWTP2-A and B, the ingoing VS content was substantially lower than in the FW plants at 2.7–2.8 g/kg. Carbohydrates represented the largest fraction of the substrate in both digesters at 43–46% of ingoing VS. In this fraction, free sugars were the most abundant (20 and 22% in WWTP2-A and B, respectively) followed by hemicellulose at 14% and cellulose at 9–10%. The remaining VS comprised 28–30% proteins, 15–20% fat, and 10% lignin-like structures (Fig. 5C). As in FW1, fat and free sugars were almost completely reduced (86–89% and 85–86%, respectively) during the degradation (20 days average HRT), while only 48–62% of protein, 47–49% of hemicellulose, and 60–63% of cellulose were degraded. Protein thus represented the largest residual macromolecule fraction in WWTP2 digesters. Hemicellulose stood out as poorly degraded in this plant versus in FW1 and FW-TD and represented the largest residual organic fraction except for proteins, at 18–23% of total VS.

### Theoretical methane potential and TMP_red_

**Fig. 6:**
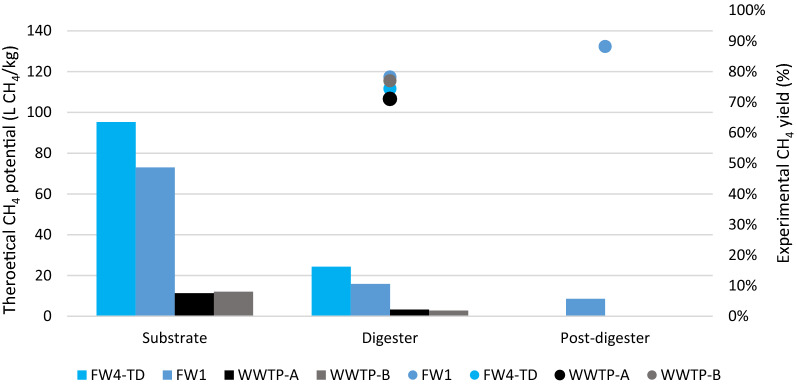
The theoretical methane potential (TMP; bars) and reduction of TMP (TMP_red_; dots), calculated according to the macromolecule content in substrate, digester, and post-digester (only applied in FW1); FW = food waste, TD = thermophilic dry digestion, WWTP = wastewater treatment plant

Based on the protein, carbohydrate, fat, and VFA contents of the substrates and digestates of the plants included in the mass balance (i.e. FW1, FW-TD, and WWTP2), the theoretical methane potentials (TMPs) of the different steps could be calculated (Fig. 6). The FW-TD substrate had the highest TMP at 95 NL CH_4_/kg ww, whereas the corresponding TMP levels for the FW1 and WWTP2 substrates were 73 NL and 11–12 NL CH_4_/kg ww (Fig. 6). Using the difference in TMP between substrate and digestate from the post-digester to calculate the TMP_red_, i.e. the fraction of substrate TMP converted into biogas, the highest value was obtained for FW1 (88%; Fig. 6), followed by FW-TD (74%) and the WWTP2 digesters (71–77%).

An interesting observation was that FW1, FW-TD, and the two WWTP2 digesters displayed VS reductions of 77, 65, and 61–63%, respectively (Table 1), while the corresponding TMP_red_ values were approximately 10% higher at 88, 74, and 71–77%, respectively (Fig. 6). Common to these digestates was that protein was the largest fraction of the digestate TMP (52–69%).

### Enzyme activity

**Fig. 7:**
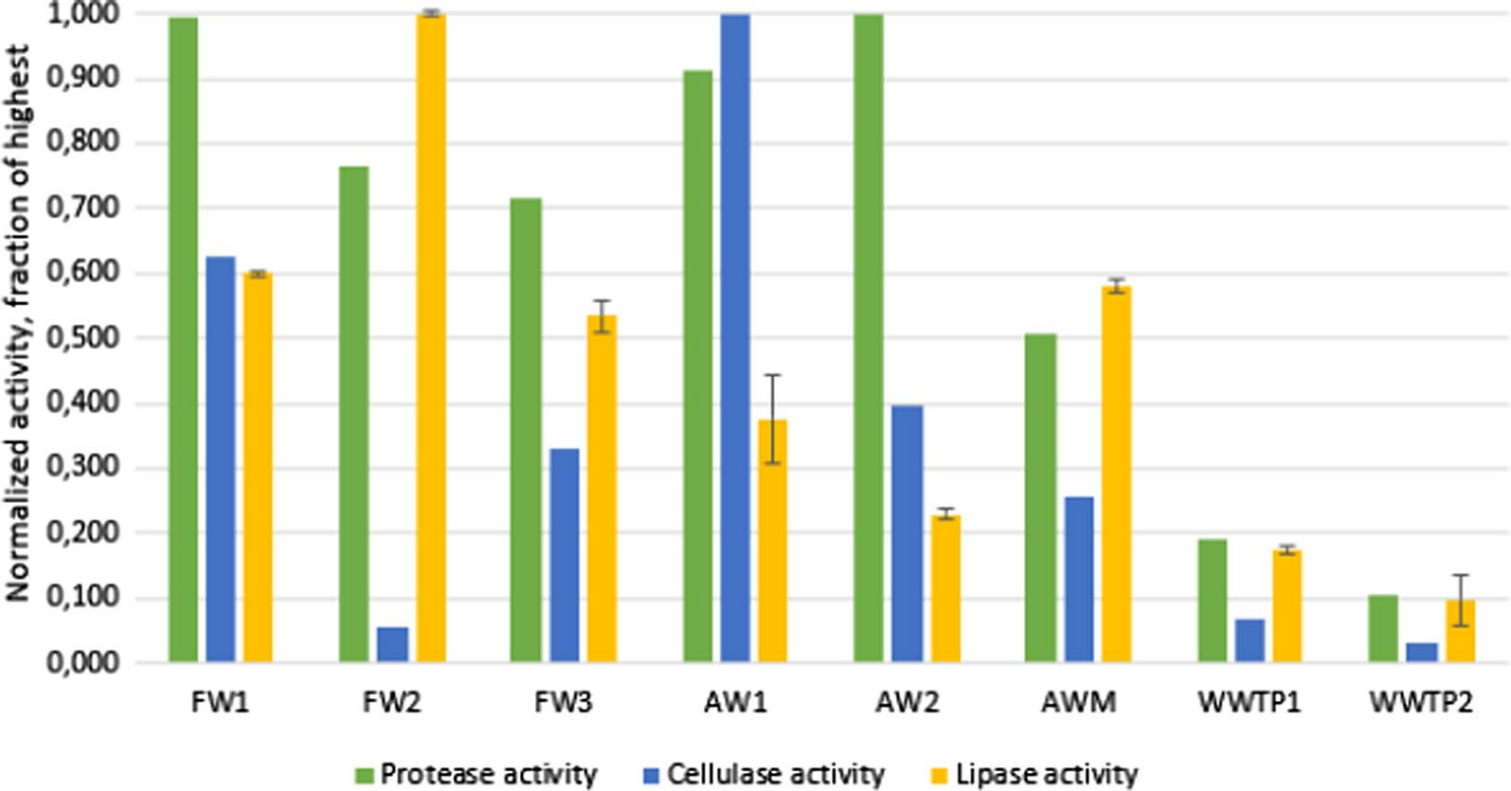
Relative enzyme activity of protease, cellulase, and lipase in digestate liquids sampled from the main digesters of full-scale biogas plants primarily operating on food waste (FW), plant-based agricultural waste (AW), agricultural waste + manure (AWM), and sewage sludge from a wastewater treatment plant (WWTP); each sample is normalized to the sample with the highest recorded value within its category; note that the activity cannot be compared between different types of enzyme activity. Error bars of normalized lipase activity values represent the same percent amplitude of error as the absolute ± 1 standard deviation has in relation to the original triplicate data

All samples presented detectable enzyme activity for all assayed activities. The various determined activities were the result of the activity of a mixture of many different enzymes having the same catalytic activity, produced by many different microorganisms (e.g., various proteases), but having different catalytic efficiencies. Considering all the investigated biogas plants, the relative protease activity was generally high in FW and AW reactors, i.e. 0.5–1 in normalized activity (Fig. 7). The highest relative protease activities were observed in FW1, AW1, and AW2 at 0.91–1 and the lowest in AWM, WWP1, and WWTP2 at 0.03–0.26 (Fig. 7).

The cellulase activity varied considerably among the plants (Fig. 7). The highest relative activity was seen in AW1 (1.0 relative activity), which had over double the activity of almost all other digesters (except for FW1 at 0.63 relative activity). Again, the WWTPs displayed the lowest activities (0.03–0.07). Notably, the cellulase activity in FW2 was also very low (0.06), much lower than in the other FW digesters (0.33–0.63).

Regarding lipase activity, FW2 displayed the highest relative activity (Fig. 7), whereas the WWTPs again gave the lowest values (0.10–0.18). Also, the two AW plants displayed lower relative activity (0.23–0.38) than did the FW reactors, while AWM (0.58) had lipase activity similar to that of FW1 and FW3.

### Viscosity, EPS and SMP

**Fig. 8:**
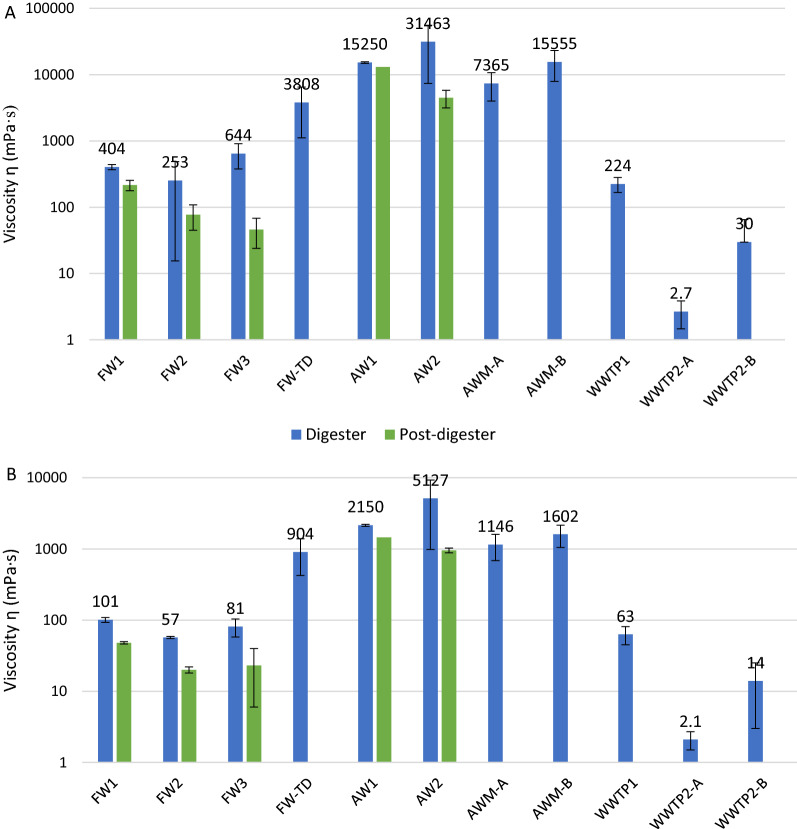
Viscosity (log mPa·s) for digesters (blue) and post-digesters (green) at shear rates of A) 20/s and B) 100/s; only one measurement was available for AW1

The viscosity in the main digesters varied greatly among the studied plants and was generally much higher at the lower shear rate of 20/s than at 100/s. Also, plants operating on AW displayed much higher viscosities than did those using other processes, i.e. 7370–31,500 mPa·s at a shear rate of 20/s and 1150–5130 mPa·s at 100/s (Fig. 8). Rheological characterization was, however, more challenging for these fibre-rich digestates, yielding large standard deviations (Fig. 8). Notably, at both investigated shear rates, the viscosity of the AW1 and AW2 digestates was higher than that of the FW-TD digestate, i.e. > 15,250 ± 450 versus 3810 ± 2700 mPa·s at 20/s, and > 2150 ± 100 versus 900 ± 500 mPa·s at 100/s (Fig. 8), even though FW-TD was operating at a higher TS (11.6% vs. 8.6–10.0%; Table 1). The viscosity of the WWTP digestates at a shear rate of 100/s was 2.1–63 mPa·s, whereas that of the FW1–FW3 digestates was 60–100 mPa·s. In all plants except WWTP2-A, the digester materials clearly acted as non-Newtonian shear-thinning fluids, giving reduced viscosity at higher shear rates.

Concentrations of extracellular polymeric substances (EPS) were generally low in the WWTPs (50–60 mg/L), and significantly higher in FW-TD and AW2 (560 and 570 mg/L, respectively; Additional file 2). In all plants the EPS concentrations decreased during post-digestion. Soluble microbial products (SMP) were more abundant, i.e. 70–1780 mg/L in most plants, whereas FW-TD and AW2 again had the highest values of 6500 and 3600 mg/L, respectively (Additional file 2). No clear pattern was seen regarding SMP concentrations when comparing main and post-digesters.

### Statistical evaluation

**Fig. 9:**
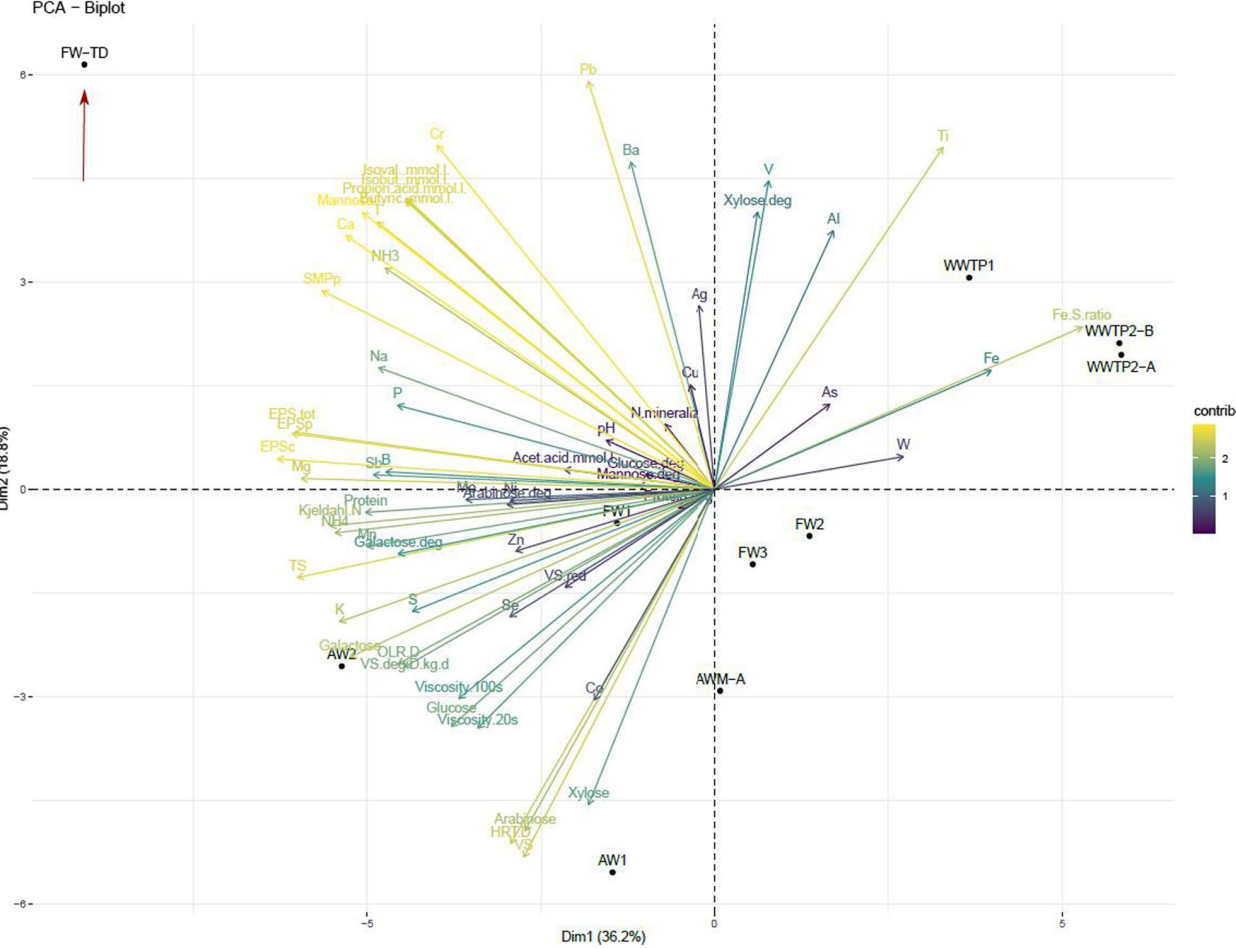
PCA of all plants, including all complete datasets. The red arrow indicates where to find FW-TD

All data from the main digesters of the biogas plants (Additional File 1) were evaluated using principal component analysis (PCA; Fig. 9). The two first principal components accounted for 55% of the variation in the data, with the most important parameters being EPS, TS, and Mg for PC1 and Pb, VS, and HRT for PC2. Also including the third principal component accounted for 70% of the data (Additional file 3), and for this component, the most important parameters were Cu and Zn, followed by W, Se, and Mo. This analysis suggests that residual sugars, such as arabinose, glucose, and galactose, constitute the main explanation for the grouping of the AW digesters (Fig. 9). Viscosity evidently also played a role, appearing to correlate positively to the residual sugars.

The reason why FW-TD is distinct from the remaining digesters seems primarily related to the concentrations of VFAs, NH_3_, and Ca in the digestate, and to the residual concentration of mannose. In the WWTPs, the Fe/S and Fe contents are important factors. Plotting PC1 with PC3 reveals that trace element concentrations are important in distinguishing FW2 and to some extent also FW-TD from the other digesters (Additional file 3).

All data in this study are based on single samples from full-scale biogas plants and there is a risk that some variations in the results is caused by natural variations or analytical measurement uncertainty. However, the dataset includes nine different full-scale plants, and all samples were retrieved under representative conditions at the plants by instructed operators, the data are therefore used for statistical analyses. Using scatter-plots to further evaluate correlations between parameters showed that VS reduction correlated positively to the degradation efficiencies of galactose and arabinose (*p* < 0.1) and to the degradation efficiencies of glucose and mannose (*p* < 0.05; for *r* values, see Additional file 4). VS reduction also correlated positively to the amount of Co and Ni in the main digester (*p* < 0.05; Additional file 4). Degradation of both galactose and arabinose correlated positively to Na concentration (*p* < 0.05), and high degradation of VS per day correlated to high protease and cellulase activity (*p* < 0.5 and 0.01, respectively; Additional file 4). Protease activity correlated positively to high ammonium and Kjeldahl-N content in the digestates (*p* < 0.01), while lipase activity correlated positively to fat content (*p* < 0.01) and negatively to Fe concentration (*p* < 0.05; Additional Files 4 and 5). Acetate formation displayed a strong positive correlation to residual fat content (*p* < 0.01), while RMP per kg ww displayed significant positive correlations to galactose (*p* < 0.01), glucose (*p* < 0.01), arabinose (*p* < 0.05), and protein (*p* < 0.01). Moreover, high RMP correlated to high Kjeldahl-N and high viscosity, but displayed a negative correlation to the Fe content in the digesters (*p* < 0.01, Additional files 4 and 5).

In this study, high viscosity was clearly correlated to high VS, but not TS, content in the digesters (*p* < 0.01 and 0.05 at shear rates of 20/s and 100/s, respectively), and in terms of VS content, high viscosity also correlated positively to arabinose (*p* < 0.01 and 0.05 at shear rates of 20/s and 100/s, respectively; Additional file 4) and extracellular polymeric substances as proteins (EPSp) (*p* < 0.5); moreover, there was a strong positive correlation at both shear rates to K, Mn, and Mg (Additional file 4).

## Discussion

### What remains and why?

The chemical compositions of the organic residues in digestate have been evaluated and discussed in a number of publications, mainly focusing on the stability of the digestate relative to its use as soil amendment/biofertilizer [17–19]. These studies suggest that carbohydrate, fat, and protein structures are all reduced during the AD process, leaving mainly more stable aliphatic and, to some extent, aromatic structures originating from fibre in the digestate. However, little is known about the correlations between operational parameters and the composition of residual organic matter in digestate, or about the correlation between digestate composition and residual methane production. Cluster analysis showed that the substrate category determined the clustering of the biogas plants in terms of chemical and operational parameters, although FW2 was an exception, clustering with AW plants rather than other FW plants (Fig. 1). Clustering according to substrate category was expected as the composition and chemistry of the feedstock should affect both operational management and the character of the resulting digestate, and similar categorization has been observed previously for microbial gene expression [20, 21]. These results are also supported by a previous study conducting a non-target analysis of the dissolved organic matter composition in full-scale biogas reactors, showing that digestates originating from the AD of sewage sludge had characteristics distinctly different from those of digestates from co-digestion processes digesting different mixtures of organic wastes [22]. In that study, the operational temperature of the co-digestion reactors was also reported as a likely important parameter regarding differences in the residual dissolved organic matter characteristics. The study also showed that proteins were enriched in the digestates relative to carbohydrates [22].

Below follows a detailed discussion of the fate of different macromolecules relative to operational parameters and substrate category.

#### Protein

In line with the findings of Shakeri Yekta et al. [23], protein was the most abundant macromolecule in all investigated digestates, except that from AW1. Moreover, the in-depth mass balance study of three of the plants (i.e. FW1, FW-TD, and WWTP2) showed that the protein fraction of VS increased from substrate to digestate, while the fat and carbohydrate fractions were degraded to a larger extent. The degradation efficiencies of the biogas plants digesting AW displayed large variation, with protein degradation efficiencies ranging from 27–44% in AWM to about 55% in AW2 (Fig. 3). The protein degradation in AW1, determined to be 100%, however, is likely incorrect. This plant (AW1) received a larger proportion of easily degradable carbohydrates than did the other investigated plants (Table 2), resulting in high overall VS reduction, and since the estimation of Y_prot_ (protein from microbial growth) is based on VS reduction, the calculation of residual protein might be underestimated (see Sect. 4.5). In contrast, AWM displayed poor hydrolysis of the protein, which could partly be connected to the type of substrate, which mainly comprised manure (67%; Table 1). In a previous study of the AD of pig manure, similarly low values for protein degradation efficiency were obtained (average 40% [24]). In line with this, a study investigating the AD of manure showed that the residual gas production from digestates mainly derived from the cellulose and hemicellulose fraction and that the protein fraction remained undigested [25]. Low protein degradation was also observed in the main digesters of the FW1 and FW2 processes (21% and 32%, respectively, calculated from values in Fig. 2), possibly caused by the slaughterhouse waste used as substrate in these plants, which likely contained recalcitrant protein fractions such as collagens which is known to be difficult to hydrolyse [26]. Consequently, proteins appear to have the highest potential for increased biogas production in digestates from different types of substrate categories. Higher protein hydrolysis, i.e. degradation efficiency would also improve the digestate quality as it would result in high levels of plant-available ammonium (NH_4_^+^) as well as potentially decreased emissions of GHG (i.e. CH_4_) from digestate storage [25]. The low degradation and higher VS fraction of protein could partly be explained by the fact that the outgoing proteins to some extent originated from recalcitrant microbial biomass (Y_prot_) produced during the anaerobic degradation of VS. It is well known that substrates dominated by bacterial biomass, such as secondary sludge from WWTP, are rich in proteins and quite recalcitrant and therefore difficult to hydrolyse [27, 28]. Similarly, the WWTPs in this study, which received secondary sludge as substrate (ca. 10–30% of ingoing material), displayed even lower protein degradation than did the other investigated biogas plants, as well as low VS reduction of 45–50%. Comparing the protein degradation efficiencies of the sewage sludge digesters (i.e. WWTPs) interestingly revealed that WWTP1 and WWTP2-B, both of which received grease separator sludge in addition to mixed sludge, had similar protein degradation efficiencies of 59% and 62%, respectively (Fig. 3). WWTP2-A, which received only mixed sludge, had a relatively lower protein degradation efficiency of 44% even though the amount of primary sludge going into this digester was higher at the time of sampling (27% vs. 17 and 12% in WWTP2-A and -B, respectively, based on TS). This indicates that the fat additions could have positive effects, promoting the more efficient degradation of the WWTP substrate. This could be due to a synergistic co-digestion effect observed for other substrates, perhaps generated by increased activity of the microbial community through improved environmental prerequisites for the active microorganisms [29, 30]. Another explanation could be that adding easily degradable substrate increased the growth and/or activity of the microorganisms in this environment. This phenomenon has previously been observed in several studies of the microbial activity and degradation of organic matter in soil, and has in that field been referred to as the “priming effect” [31].

Hydrolysis of protein releases ammonium and ammonia, which can be problematic as ammonia-induced process disturbances in continuous digesters have been observed over a wide range of ammonia concentrations, i.e. 0.2–1.5 g/L NH_3_-N (reviewed by Capson-Tojo et al. [32]). The disturbances are typically more pronounced at higher operational temperatures, as this causes the level of free ammonia to increase. Ammonia inhibition could be one reason for the VFA accumulation and low VS reduction observed in FW-TD (> 8 g/L VFAs, > 0.7 g/kg NH_3_-N), and previous studies have shown a correlation between these two parameters (reviewed by Capson-Tojo et al. [32]). This high VFA accumulation was probably one reason why FW-TD did not cluster with any of the other plants in the clustering analysis, although the thermophilic conditions and higher TS content of the digestate than in the other plants likely also contributed. It has been observed that ammonia inhibition can cause the accumulation of acetate and, in more severe inhibition cases, propionate [10, 33], the latter being the major acid produced in FW-TD. In this study, NH_4_^+^-N production correlated positively to the accumulation of acetate in the different reactors (*p* < 0.05).

Another plant with high VFA concentration was FW2, which displayed the lowest VS reduction of all studied plants. Of special interest here was the difference in performance between FW1 and FW2. Both processes treated similar substrates (although FW2 with a slightly shorter HRT) and had similar NH_3_-N levels, but still FW2 showed indications of instability with higher levels of VFAs as well as lower VS reduction compared with FW1. The metal analysis revealed that the level of the trace metal cobalt (Co) was low in FW2 (0.2 mg/kg), which is close to what has previously been determined to be critical for FW digestion [33] and lower than that in the other FW plants (0.7–0.9 mg/kg). Similarly, the Ni and Se contents were relatively low in FW2 (0.3 mg/kg and 0.04 mg/kg, respectively). The importance of Co, Ni, and Se for methanogenesis and SAO is well established [34], and the low levels of these elements in FW2 suggest a lack of trace elements, resulting in elevated VFA levels in the FW2 main digester. The level of Co in FW-TD was also low (0.1 mg/kg), which might be one factor contributing to the observed high levels of VFAs in this digester, particularly considering that requirements for trace elements (including Co) appear to be even higher in thermophilic conditions [35] and at high NH_4_-N concentrations [33]. The fact that the Co and Ni contents affect the overall digestion efficiency was also supported by the positive correlation of Co and Ni to VS reduction (*p* < 0.05, *r* > 0.7) found in this study. The measured protease activity did not correlate to the protein degradation efficiency, which is likely explained by the build-up of protein-rich microbial biomass (Y_prot_) masking the protein degradation of the substrate. This assumption is supported by the correlation between protease activity and the concentration of nitrogen, in both NH_4_-N and Kjeldahl-N forms, released during amino acid degradation, which represents an indirect measure of protein degradation.

In summary, considering the protein concentration across different plants and from substrate to post-digester in this study, post-digestion results in substantially increased protein hydrolysis and hence degradation. Processes not applying post-digestion, for example, in the WWTP and AWM plants, had low protein degradation compared with the other plants. This could be because, first, both fat and free sugars are quickly degraded, while protein degradation is relatively slow, particularly under acidifying conditions [36], and, second, the microbial biomass produced during substrate degradation is rich in proteins and could be considered a post-digester substrate. However, accessing the organic material of the microbial biomass is challenging, and requires treatment before post-digestion.

#### Carbohydrates

The AW plants displayed high VS reduction (78 and 77% for AW1 and AW2, respectively), which could partly be explained by the relatively long HRTs applied by this plant category. AWM, which used a relatively shorter HRT, displayed a lower VS reduction of 62%. Long HRTs are generally necessary when treating AW rich in recalcitrant lignocellulosic materials which are slowly hydrolysed [25, 37, 38]. In a survey of 21 full-scale plants operating on AW, only two plants used HRTs under 60 days, and the average digestion time was about 100 days [15]. In a German survey of biogas plants operating primarily on plant-based AW (i.e. 55–100% crop residues), the HRTs were 46–191 days (*n* = 24, mean = 98 days; [38]). However, a study of Danish biogas plants operating mainly (> 70%) on manure demonstrated that the HRTs were typically shorter than in digesters running on plant-based substrates only, with HRTs of 18–62 days (*n* = 11, mean = 32 days; Supplementary Material in Hamelin et al. [37]). Even though lignocellulose-rich materials are known to be difficult to hydrolyse and hence degrade during AD, the VS reduction in the plant-based digesters was similar to that in FW1 and FW3, showing that high VS reduction can be obtained with long HRTs. However, the benefits of prolonging the HRT for enhanced degradation should be weighed against the drawbacks of inefficient biogas production per time unit, which reduces plant profitability.

In all plants, VS reduction correlated to the degradation of sugars (e.g., galactose, glucose, mannose, and arabinose) as well as to the cellulase activity per g VS (Additional file 4). An exception to this was FW2, which displayed very low degradation of sugars (5%) and was among the lowest in cellulase activity, i.e. just 6% of the highest activity (Fig. 7). The sugar content of the substrate in this plant was much lower than that in the other FW plants, for example, 4 g/kg versus 40–46 g/kg in FW1 and FW3, whereas the level of VFA was higher at 160 mmol/L versus 50–60 mmol/L in FW1 and FW3 (Additional file 1). Combined, this indicates that pre-hydrolysis of the sugars in the substrate occurred before entering the digester. This conclusion is also supported by the VFA profile of the substrate mixture of FW2, as a high ratio of acetate and butyrate combined with a low pH (5.4) is characteristic of dark fermentation (i.e. H_2_ production [39]).

The difference in carbohydrate degradation between the biogas plants (except FW2) was likely related to their origin and composition. AW1 and AW2 primarily digested lignocellulosic-rich materials (e.g., crop silage, corn silage, cereals, and grain chaff), the carbohydrate degradability of which is limited due to their recalcitrant hemicellulose–lignin structures (reviewed by Carrere et al. [40]). In addition, a large fraction of the substrate in AWM was manure, which also contains recalcitrant fibre fractions remaining after feed digestion as well as bedding materials [24], which could explain the low degradation efficiency of carbohydrates in this plant (71%) versus the others (91–92%). Degradation of free or complexed xylose did not correlate with VS reduction, but as this sugar monomer is one of the main components of hemicellulose [41], this result is likely related to the recalcitrance of the lignocellulosic substrates discussed above. The silage at AW1 was rich in xylose (185 g/kg), but despite the low degradation efficiency of xylose at this plant, the VS reduction in the main digester was relatively high at 67%, likely due to the easily degradable sugars in the starch slurry. The decrease in lignin-like structures observed in FW1, WWTP2, and FW-TD (23–63%) was unexpected, as native lignin is considered highly recalcitrant under anaerobic conditions. One explanation could be overestimation of the ADL fraction in the lignin analysis, as a high content of free lipids (> 10% of TS) could interfere with the analysis [42].

The residual amounts of cellulose and hemicellulose detected in the detailed investigation of the three biogas plants differed somewhat, as shown in Fig. 5. The fact that the cellulose content was higher than the hemicellulose content in the substrate of FW1, while the opposite was seen in the FW-TD substrate, was probably partly due to the 2% (volumetric) addition of garden waste to FW-TD (Table 2), and partly because this plant used brown paper bags to collect the FW (FW1 used plastic bags for collecting the organic FW). When comparing the FW digesters, the degradation efficiencies of hemicellulose were similar at 70–76%, while the cellulose degradation was much lower at 55% in FW-TD versus 82% in FW1. The reason for the lower degradation of cellulose could be related to the high ammonia content in the FW-TD digester resulting from the high temperature and high ammonium-nitrogen concentration (Table 1). In previous studies, a high ammonia concentration of 0.3–0.41 g/L was found to reduce cellulose degradation efficiency [43, 44].

#### Fat

In general, fat degradation was efficient in all plants for which data were available. The degradation of fat can be challenging at high loads and lead to problems with foaming, floatation, and inhibition by long-chain fatty acids (LCFAs; [45, 46]). In this study, FW1 had the highest substrate fat content at 32% of total VS, yet with low concentrations of fat in the digestate. The efficient fat degradation could relate to a high abundance of syntrophic β-oxidizing bacteria and Cloacimonadota, which have been correlated to efficient degradation of lipids and long-chain fatty acids, respectively [47, 48]. In addition, FW digesters might have a microbial community better adapted to maintaining the low partial pressure of hydrogen required for efficient lipid oxidation [49], as high ammonia concentrations result in more abundant or active hydrogenotrophic methanogens that via syntrophic acetate-oxidation produce methane through hydrogen consumption.

From a physical/mechanical perspective, the often higher TS resulting from co-digestion with FW compared with, for example, sewage sludge digestion, provides more surface area for the lipids to adhere to, and could thus lead to better mixing with the fats [50] and improved lipid accessibility for the microorganisms. Furthermore, co-digestion plants typically have mandatory pasteurization of their substrate mixtures (as in FW1–FW3 in this study), in which heating, melting, and dissolution of the fat fractions result in better mixing with the remaining substrate and thereby improved degradation.

The fat degradation efficiency of the FW2 main digester was notably low at 11%, but this might, as discussed in "Protein", be related to the low levels of trace elements and the combination of high levels of ammonia and VFAs that strongly indicates inhibition of the methanogens. Synergetic co-inhibition caused by LCFAs and high ammonia has previously been observed by Tian et al. [51], who argued high ammonia led to the accumulation of hydrogen and acetate, in turn rendering the β-oxidation pathway thermodynamically unfavourable, concomitantly with an accumulation of LCFAs leading to inhibition.

Lipase activity correlated positively to residual fat content in the main digesters (Fig. 8; Additional file 4), and this was particularly evident in FW2, which displayed the highest lipase activity and had a fat content over three times higher than those of the other FW processes. Since the raw fat analysis includes both lipids and the LCFAs that are soluble in petrol (used during the extraction), it cannot be fully determined that there was an accumulation of LCFA, but, based on the enzyme activity and the poor performance of the digester, this was likely the case. Unfortunately, high lipase activity in a system in which LCFA degradation is hampered increases the risk of inhibition from LCFAs, as observed previously [52].

In addition, correlation analysis revealed a negative correlation between Fe concentration and lipase activity (*r* = –0.7; Additional file 4), but whether Fe has a direct effect on lipase activity or whether the correlation is because of an indirect relationship is unclear and will require further investigation.

### Viscosity, EPS, and SMP

Low VS reduction results in higher VS content in the digestate, and in the present study high viscosity was clearly correlated to high VS content (*r* = 0.74 at a shear rate of 100/s; Additional file 4). The fact that higher viscosity was observed in AW1 and AW2 despite having similar or lower VS contents compared with FW-TD indicates that the composition rather than concentration of the VS is important when comparing digesters operating on different substrates. High viscosity can, apart from increasing the power demand [53], also negatively affect the mixing efficiency of digesters (reviewed by Lindmark et al. [54]), causing the formation of dead zones, sedimentation, and floating layers and ultimately leading to reduced degradation efficiency of the biogas process ([55]). In addition to VS content, a positive correlation between viscosity and the presence of EPS/EPS_p_ and of cations (i.e. K, Mg, and Mn) could be identified in the biogas plants. Both cations and EPS have previously been found to increase viscosity, as the cations bridge and strengthen the polymer network, thus affecting the viscosity accordingly [56–58]. In line with this, there was a positive correlation of viscosity to arabinose (likely in the form of plant polysaccharides) at both investigated shear rates, with the highest content of arabinose being seen in AW1, followed by AW2 and AWM (Additional file 1). Lastly, the viscosity in the post-digesters was lower than in the main digesters (Fig. 8), possibly because the carbohydrate fraction of the EPS (EPSc) decreased due to the degradation of these molecules in the first digestion step, but not enough post-digesters were sampled to confirm this statistically.

### RMP, TMP, and TMP_red_

The residual methane potential (RMP) is a good quantitative measure of how much of the remaining organic material in the digestate could actually contribute to increased biogas production, although it only shows the methane production that could be obtained without any further treatment [59]. Previous studies of RMP from various digestates report values of 20–240 mL CH_4_/g VS, in extreme cases corresponding to as much as 50% of the total biogas production, depending on the type of substrate and operation [15, 16, 60]. Several studies show a positive correlation between RMP and OLR and a negative correlation between RMP and HRT [4, 16]. In this study, RMP correlated positively to the OLR of the digester (*p* < 0.05; Additional file 4), while there was no clear negative correlation between RMP and HRT. The plants with both among the longest HRT (AW2) and shortest HRT (WWTP1A-B) had low RMPs, and the plant with the longest HRT (AW1) had among the highest RMPs. This was likely explained by the characteristics of the different substrates used; in contrast, Ruile et al. [16] included only agricultural digesters in their study. Instead, in this study, RMP (measured in L CH_4_/kg) was positively correlated to VS content.

Comparing the specific RMPs (L CH_4_/kg VS), AW2 and the WWTPs displayed low potentials, which indicates that the quality of the outgoing VS from these plants was lower than that of the other plants in terms of potential biogas production. It also suggests that efforts to increase the gas production from these plants only by increasing the HRT would not likely be worthwhile. In addition, the low protein degradation efficiency in these plants (48–62%), together with the relatively high residual protein content in the digestates, suggests the presence of recalcitrant protein structures, and hence that post-treatment targeting proteins would be more efficient than only increasing the HRT in accessing this potential gas production. This conclusion is supported by the fact that only 6–13% of the theoretical methane potential (TMP) was obtained during the RMP tests in the WWTPs, corresponding to 0.2–1.2 L of additional CH_4_ per kg substrate. In all digestates, except that in AW1, protein was the largest contributor to residual TMP, emphasizing the importance of targeting this macromolecule for increased biogas production.

WWTP1 had relatively high RMP and low VS reduction compared with WWTP2-A and -B, which were also treating grease separator sludge (55% vs. 63 and 61% in WWTP2-A and -B), further supporting the conclusion in Sect. 3.1.1 that protein degradation was more efficient when fat was added as a co-substrate.

Among the plants with higher specific RMP, FW2 and FW-TD both had high VFA contents and low VS degradation. FW-TD also contained a high level of crude fat relative to FW1 (4.1 vs. 1.5 g), which is also correlated to high gas potential and likely contributed to the high RMP in this case. However, the reason for this is partly related to process instabilities, meaning that the RMPs would likely be reduced if process operation was adjusted (i.e. adding trace elements or lowering the digestion temperature in the thermophilic digester). AWM had the highest RMP (170 mL CH_4_/g VS) and this suggests that a longer HRT could be a suitable measure to enhance the methane production.

In FW1, FW2, FW-TD, and AWM (Fig. 6 and corresponding values for FW2 and AWM, not shown), only 40–46% of the TMP was obtained during the RMP tests (Fig. 4B), even though they were run for about 100 days. In AWM and FW-TD, this resulted in 8.6 and 9.1 NL CH_4_/kg substrate, respectively, indicating that increasing the digestion time or adding a post-digestion step should be considered, although for plants with high VFAs (i.e. FW-TD and FW2), improving the performance of the main digestion to prevent VFA and LCFA accumulation should be the primary focus. Post-digestion could be a relatively easy measure to extract more methane from existing substrate and, in light of methane as a potent GHG, it would also be a way to reduce methane emissions during digestate storage before land application. Such emissions can be substantial, as demonstrated in a study finding that digestate emissions at a manure and FW co-digestion plant without post-digestion amounted to 12% of the plant’s yearly methane production [60]. Similarly, a survey of manure-based digesters showed that an estimated increase in methane production of about 20% could be achieved by prolonging the HRT from 30 to 60 days [37].

The reduction in theoretical methane potential (TMP_red_) measures how much of the ingoing TMP is harvested in the biogas process, i.e. a high TMP_red_ indicates an efficient process. Interestingly, the TMP_red_ was higher than the VS reduction in several of the processes included in the mass balance, likely because fat, proteins, and carbohydrates have different gas yields [5]. For example, the TMP_red_ of FW1 was 88% compared with a VS reduction of 77%, which is related to the fact that almost all the fat, which carries the highest methane production potential per g, was degraded, while some proteins and carbohydrates remained (harbouring lower methane production potential per g). From a methane production perspective, this somewhat lowers the incentive to further treat and digest the digestate in FW1, since the additional methane it would produce (a maximum of 12% of the substrate methane potential) might not compensate for the added costs. In AWM, on the other hand, TMP_red_ was lower than the VS reduction (55% vs. 62%), suggesting that a significant amount of gas could yet be extracted. In FW-TD, the TMP_red_ reached only 74%, indicating that the process disturbance observed and/or lack of post-digestion limited the degree of degradation and that up to 26% more gas could theoretically be produced from the substrate.

In summary, when high residual organic content and high RMP are consequences of process instability and VFA accumulation, the first action to take would be to pinpoint the reason for the instability (e.g., lack of trace elements or ammonia inhibition) and adjust the process operation accordingly. If the digestate still has a high RMP, another action could be to prolong the digestion time of the main digester or implement post-digestion. Lastly, as clearly demonstrated in this study, many digestates covering different AD processes seem to contain recalcitrant protein fractions, either from the substrate or in microbial biomass. To better access the methane contained in this material, targeted treatment before post-digestion could be promising. In addition, it is logical to suggest post-treatment of the residual fractions rather than pre-treatment of the substrates to increase the efficiency of biogas processes, as this would avoid spending energy or chemicals to also treat the already easily accessible fractions of the substrate (e.g., fats and sugars).

## Material and methods

### Sample collection

From each biogas plant, substrate samples were collected from the digester and post-digester (if applicable). The main operational conditions, substrate composition, and sampling points are summarized in Table 1. At least 2 kg of the solid substrates and 4 L of the sludges were sampled, and the representativeness of the samples was ensured in communication with plant personnel, following their sampling routines for regular process monitoring. In most cases the final substrate mix was sampled, while at some plants the individual substrate components had to be sampled separately (Table 1). After collection, the samples were stored at 4°C for a maximum of 7 days until further analysis.

### Analytical methods

For each substrate, digestate, and post-digestate, several different analyses were performed to obtain detailed information on their properties and organic matter composition: TS, VS, pH, and VFAs were all determined within a day of sampling. TS and VS were analysed according to the Swedish Standard method (SS 028113), and the pH of all liquid samples was determined with a pHC2401–7 combination pH electrode (Radiometer, Copenhagen, Denmark) according to European Standard EN 12,176. The VFAs (i.e. acetic, propionic, isobutyric, n-butyric, iso-valeric, n-valeric, and isocaproic acids) of all liquid samples were analysed using gas chromatography as described earlier [61].

The following standardized analyses were performed by Eurofins Environment Testing Sweden AB (Lidköping, Sweden): total Kjeldahl nitrogen and ammonium-nitrogen (NH_4_-N; titrimetric analysis), raw protein ((Kjeldahl-N – NH_4_-N) × 6.25), elemental composition (ICP-AES, ICP-MS; see Additional file 6 for a detailed list of the elements measured), and raw fat (acid hydrolysis/gravimetric extraction). In addition, the total amount of xylose, mannose, glucose, galactose, and arabinose (bound and complexed) was determined by GC–MS.

The amount of lignin, cellulose, and hemicellulose was quantified through the analysis of non-detergent fibre (NDF), acid-detergent fibre (ADF), and acid-detergent lignin (ADL). The samples were dried for 2 h at 105 °C, after which NDF, ADF, and ADL were determined in sequence according to Van Soest et al. [42]. In short, NDF was determined by heat-treatment with heat-stable amylase, ADF was determined by acid-treatment, boiled (1 h) and filtered, and ADL was determined after treatment of residue from ADF in 72% sulfuric acid (3 h) all according to Van Soest et al. [42]. Thereafter, the amount of free sugars in each sample was estimated by subtracting hemicellulose and cellulose from the total content of all sugar monomers obtained from the analyses at Eurofins AB (described in the previous paragraph).

To determine the C/N ratio, the samples were freeze-dried and milled as described by Shakeri Yekta et al. [62], after which the C and N contents were determined using an elemental analyser according to the manufacturer’s instructions (Series II CHNS/O analyzer, Perkin Elmer, Waltham, MA, USA). Concentrations of dissolved organic carbon (DOC) were measured as described by Nordell et al. [63] (submitted).

A summary of the analysed parameters for each plant can be found in Additional file 7.

The extraction and quantification of extracellular polymeric substances (EPS) and soluble microbial products (SMP) was performed as described by Frølund et al. [64], with the modifications described by Ekstrand et al. [56]. EPS and SMP were then quantified as protein (i.e. EPSp and SMPp) or polysaccharide fractions (i.e. EPSc and SMPc) using a modified Lowry method [64] and the anthrone method [65], respectively, with a UV/VIS spectrophotometer (Ultraspec 2100 pro, Biochrom Ltd., Cambridge, UK). Bovine serum albumin (BSA) and glucose were used as protein and polysaccharide standards, respectively, to determine BSA- and glucose-equivalent concentrations of proteins and polysaccharides. All extractions and analyses were conducted in triplicate, using three subsamples from each reactor sample.

Rheological characterization of all digestates and post-digestates was performed in triplicate at 37 ± 0.2, 38 ± 0.2, 42 ± 0.2, or 55 ± 0.2 °C, corresponding to the operational temperatures of the digesters and post-digesters at the plants (Table 2). The analyses were performed using a shear rate-controlled Searle-type rotational rheometer, as described by Ekstrand et al. [56]. In short, a three-step protocol was implemented in which the shear rate was (1) increased linearly from 0 to 800/s over a period of 800 s; (2) maintained constant at 800/s for 300 s, and (3) decreased linearly from 800 to 0/s over a period of 800 s. Due to the often non-Newtonian character (i.e. a nonlinear relationship between shear rate and shear stress) of AD sludge, apparent viscosity (η) was determined at two shear rates, η_20_ at 20/s and η_100_ at 100/s. The shear rates were chosen based on the study by [66], which demonstrated local shear rates of up to 100/s in reactors mixed at 200 RPM.

### Residual methane potential

The residual methane potential (RMP) of the digestates was determined in triplicate using 1130-mL glass bottles, sealed with rubber stoppers and aluminium screw caps. First, 200 g of digestate was added to each bottle while flushing with N_2_. After sealing, the headspace gas in the bottles was exchanged with N_2_:CO_2_ (80:20) according to Holliger et al. [59]. The gas production in each bottle was determined once a week as described by Ekstrand et al. [67]. For the FW1 and WWTP1 plants (Table 2), 9 L of digestate was instead used in 12-L laboratory-scale biogas reactors; the reactors were subjected to continuous stirring (80 rmp) and the headspace was flushed with 100% N_2_. Volumetric gas production was measured online using a Ritter MilliGascounter (MGC-10, Ritter, Waldenbuch, Germany), and methane concentration was determined online using a gas sensor (BlueSens, Herten, Germany). All gas volumes were normalized to standard temperature and pressure (273.2 K and 1.01325 bar, respectively). After 100–120 days, the experiments were ended, and the RMP at that time was calculated as the volume of methane produced per gramme of VS added (NL CH_4_/g VS), as well as L CH_4_/kg.

### Enzyme activity

To separate microorganisms and large particulate matter from the digester fluid, the digestate samples collected from the main digesters at the biogas plants were initially centrifuged at low relative centrifugal force for 10 min immediately after arrival at the laboratory. The supernatant was frozen and stored at – 18 °C. For enzyme activity analyses, the samples were prepared by thawing and centrifuging 1-mL aliquots at 13,000*g* for 15 min at room temperature in 1.5-mL Eppendorf tubes. Supernatants from the same biogas plant were pooled, after which they were divided into new aliquots and stored at – 18 °C until further analysis. The assay temperature for all enzyme activities was 37 °C, and the analyses were performed in either duplicates or triplicates.

Protease activity was monitored using resorufin-labelled casein (Roche Diagnostics, Mannheim, Germany), in accordance with the manufacturer’s instructions. After 4 h of substrate/sample incubation, the remaining undigested labelled casein was precipitated with trichloroacetic acid, the supernatant was collected, and the pH was increased to 8.8. After all steps of incubation, precipitation, and pH change, the absorbance at 574 nm was registered in a 1-cm cuvette using a Biochrom Biowave diode array spectrophotometer (Biochrom Ltd, Cambridge, UK). Since the samples with remaining resorufin-labelled casein peptides absorb light in the visible wavelength range after pH change, it could also be visually confirmed that the samples displayed protease activity.

Cellulase activity was monitored in duplicates in microtiter plates using resorufin-labelled cellobioside (Markergene Technologies Inc, Eugene, OR, USA) in accordance with the manufacturer’s instructions. The fluorescence increase caused by cellulase activity was monitored for 60 min on a Clariostar plate reader (BMG Labtech, Ortenberg, Germany) using ex/em wavelengths of 545 and 600 nm and bandwidths of 20 and 40 nm, respectively.

Lipase activity was monitored in triplicate using a methylresorufin-labelled substrate (Sigma-Aldrich, Saint Louis, MO, USA) in microtiter plates in accordance with the manufacturer’s instructions. Fluorescence caused by lipase activity was monitored on a Clariostar plate reader (BMG Labtech, Ortenberg, Germany) at ex/em wavelengths of 529 nm and 600 nm and bandwidths of 20 and 40 nm, respectively, until the value of the most active sample reached the detection limit of the instrument. As a measure of reproducibility, for each triplicate the standard deviation of the final data point was calculated. Since the data were later normalized, the standard deviation was first recalculated to the plus/minus percentage of the final fluorescence value and the obtained percentage was used calculate the corresponding value of deviation of the normalized lipase activities.

For all samples and methods, the sample background absorbance or fluorescence was registered and subtracted from the assay results by treating samples the same way as assay samples, but without adding substrate. In addition, substrate autocatalysis or degradation was registered and subtracted from the assay results by running samples with only substrate in reaction buffers under the same conditions and times as the assay samples. For a relative comparison of the enzyme activity across the different plants, the activities of each enzyme (i.e. protease, cellulase, and lipase) were normalized to the highest value of each enzyme activity.

### Calculations

The amounts of organic material (i.e. proteins, fats, and sugars) in the digested samples (g/kg) were adjusted to account for the reduction in liquid reactor volume due to gas production (see Additional file 8 for calculations). The same volume reduction was also used to adjust the VS reduction, as follows:

$$VS\ red\ adj \left(\%\right)=\frac{{TS}_{out}\left(\%\right)\bullet {VS}_{out}\left(\%\right)\bullet \left(1-vol.red\left(\%\right)\right)}{{TS}_{in}\left(\%\right)\bullet {VS}_{in}\left(\%\right)},$$

where vol.red is the volume reduction, red adj is adjusted reduction.

To study the degradation efficiencies of the different organic fractions, *X*_deg_ was calculated as:

$${X}_{deg}=\frac{{X}_{in}-{X}_{out}}{{X}_{out}},$$

where _deg_ is degradation, *X* is protein, sugar, or fat, *X*_in_ is the amount (g) entering a digestion step (i.e. main or post-digester), and *X*_out_ is the amount (g) in the sludge leaving the digestion step (volume adjusted).

Nitrogen mineralization was determined in % as:

$$Nitrogen \ mineralisation \left(\%\right)=\frac{{NH}_{4}\cdot {N}_{out}-{NH}_{4}\cdot {N}_{in}}{{Kjeldahl.N}_{out}}.$$

The total amount of degraded VS (kg/m^3^·day) was calculated as OLR multiplied by VS red adj. In addition, to adjust the protein content of the digestates to account for biomass growth, an estimation of “new” biomass protein was made (*Y*_prot_). It was assumed that 0.1 g of biomass was formed for each gramme of VS degraded (10% converted to new microbial biomass), and that 50% of that formed biomass was protein [68]:

$$Degraded\ VS \left(\frac{g}{kg}\right)= {TS}_{in}\left(\%\right)\bullet {VS}_{in}\left(\%\right)\bullet 1000g\bullet VS\ red \ adj (\%)$$

$${Y}_{prot}\left(g/kg\right)=0.1\bullet 0.5\bullet Degraded\ VS \left(\frac{g}{kg}\right)$$

Finally, the residual protein (i.e. from undegraded substrate) was estimated as:

$${Protein}_{resid}\left(g/kg\right)=Protein \left(g/kg\right)-{Y}_{prot}\left(\frac{g}{kg}\right),$$

where _resid_ is residual.

Theoretical methane potential (TMP) per kg added material was calculated according to the content (g/kg) of each macromolecule in the substrate, digester, and post-digester, and to the content of VFA. The TMP of the macromolecules was calculated according to the Buswell formula, assuming 0.42 L CH_4_/g carbohydrate, 1.01 L CH_4_/g fat, and 0.50 L CH_4_/g protein [5], as:

$$TMP\left(\frac{L{ CH}_{4}}{kg}\right)=0.42 L\bullet \frac{g\ carbohydrate}{kg}+1.01 L\bullet \frac{g\ fat}{kg}+0.50 L\bullet \frac{g\ protein}{kg}$$

while the TMP from VFAs was calculated using molar equivalents of n CH_4_/n VFA according to Schink [49]. Furthermore, to estimate how much of the TMP was obtained in each digestion step, TMP reduction (TMP_red_) was calculated as:

$${TMP}_{red}\left(\% of substrate\right)=\left(\frac{{ TMP}_{substrate}-{ TMP}_{digestion step}}{{ TMP}_{substrate}}\right).$$

Calculations of free ammonia nitrogen (NH_3_-N) were performed using measured ammonium-nitrogen (NH_4_^+^-N), pH, and temperature according to Hansen et al. [69].

### Statistical evaluation

To explore possible correlations between measured and calculated parameters, several statistical methods were applied. All statistical analyses were performed using the software R [70].

Hierarchical clustering analysis was performed using Ward’s minimum variance method [71] on scaled data. All complete datasets for the main digesters as well as residual fractions for the last digestion step were included, i.e. residual organic fractions, metals, operational data (e.g., OLR, HRT, temperature), EPS and SMP concentrations, apparent viscosity, ammonium and ammonia concentrations, and all calculated parameters listed above. Parameters not included (due to missing data for at least one plant) were DOC, SMPc, fat content and degradation, C/N ratio, and enzyme activities. PCA was performed on scaled and centred data from the same dataset as was listed for the hierarchical cluster analysis, and the result was visualized using the R package factoextra [72].

To determine pairwise correlations between parameters, scatter-plots for selected parameters were plotted together with the linear regression and the corresponding Pearson coefficient (*r*) and *p*-value.

## Conclusions

This study focused on residual methane potential and the destiny of macromolecules in different full-scale biogas processes, from substrate to digestate. Our results showed that:

- Protein was the most abundant macromolecule in the digestates from plants operating on FW and sewage sludge (3–21 g/kg), while free sugars and fat were efficiently degraded
- High residual protein content was partly coupled to recalcitrant protein, but also to the formation of microbial biomass during substrate degradation
- Unstable digestion processes (i.e. high total concentration of volatile fatty acids and low volatile solids [VS] reduction) due to ammonia inhibition (> 0.7 mg NH_3_-N/kg), partly caused by digestion at elevated temperatures and/or deficiencies of trace elements (mainly cobalt), led to the accumulation of VFAs and to high RMP in the digestates
- Co-digestion of sewage sludge with fat increased protein degradation efficiency with 18%, possibly through the mechanism referred to as priming

Furthermore, theoretical gas yields (TMP_red_) were calculated based on the contents of macromolecules in the substrates and digestates. This parameter gave a more accurate assessment of the overall biogas efficiency compared with VS degradation, as it took the gas potential of the different types of VS into account. TMP_red_ was approximately 10%-units higher compared to VS reduction since it considered that fat (which were almost completely degraded) holds a higher gas potential than for example carbohydrates. Together with the RMP, which is related to the degradability of the remaining VS, different strategies to access the gas potential of the residual fractions could be formulated:

- To improve biogas yields of protein-rich digestates, post-treatment prior to post-digestion is desirable, as the microbial biomass formed during AD would be targeted as well. Post-treatment should focus on methods directed at disrupting microbial biomass and recalcitrant protein structures
- For improved digestion where TMP is high, for example, with high residual content of carbohydrates, extending HRTs by, for example, post-digestion would be suitable to reduce RMP and thus limit GHG emissions during digestate storage and application
- Use of trace element supplementation may be necessary to obtain a stable process, especially when operating at higher temperatures (i.e. 55 °C)

## Supplementary Information

**Additional file 1.** Results from the chemical analyses for all sampling points

**Additional file 2.** Concentration of extracellular polymeric substances and soluble microbial

**Additional file 3.** Biplot of PC1 to PC3 from the principal component analysis of all complete datasets

**Additional file 4.** Pearson coefficients for significant correlations of selected parameters

**Additional file 5.** Scatter-plot for the correlation of Fe to lipase activity and to RMP

**Additional file 6.** List of elements analysed (ICP-AES, ICP-MS) for elemental composition of substrate, digestate and post-digestates

**Additional file 7.** A summary of all parameters that were analysed for each full-scale biogas plant

**Additional file 8.** Calculation of the amount of organic material in the digested samples

## Data Availability

The datasets supporting the conclusions of this article are included within the article and its additional files.
