# Supplementary material for: Identifying targets for increased biogas production through chemical and organic matter characterization of digestate from full-scale biogas plants: what remains and why?
Source: Biotechnol Biofuels Bioprod. 2022 Feb 10;15:16. doi: 10.1186/s13068-022-02103-3 (PMC8830174; doi:10.1186/s13068-022-02103-3)
Supplement: Supplementary file 2 — Additional file 2. Concentration of extracellular polymeric substances and soluble microbial [file 13068_2022_2103_MOESM2_ESM.docx]

## Additional File 2 - Concentration of extracellular polymeric substances and soluble microbial products


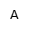

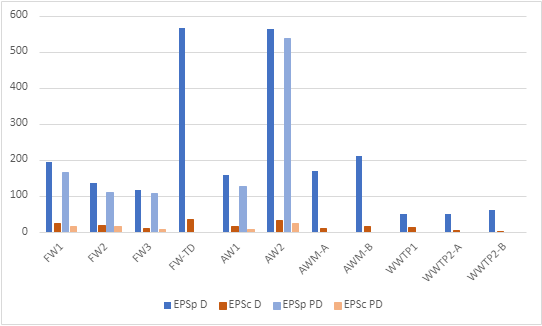


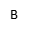

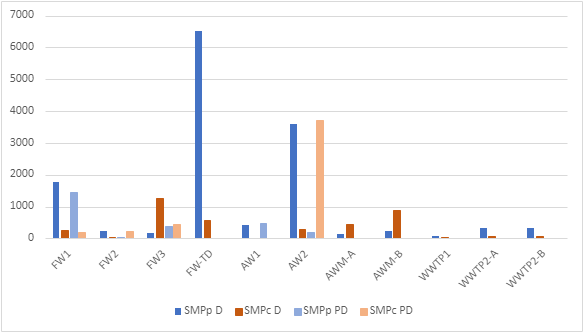


*Fig. 1 The concentration of A)* *extracellular polymeric substances as proteins (EPSp) as and carbohydrates (EPSc), and B) soluble microbial products as proteins (SMPp) and carbohydrates (SMPc) in main digesters (D) and post digesters (PD; when present) at full-scale biogas plants. FW = food waste, TD = thermophilic dry digestion, AW = plant-based agricultural waste, AWM = agricultural waste + manure, WWTP = wastewater treatment plant*
