# Supplementary material for: Identifying targets for increased biogas production through chemical and organic matter characterization of digestate from full-scale biogas plants: what remains and why?
Source: Biotechnol Biofuels Bioprod. 2022 Feb 10;15:16. doi: 10.1186/s13068-022-02103-3 (PMC8830174; doi:10.1186/s13068-022-02103-3)
Supplement: Supplementary file 3 — Additional file 3. Biplot of PC1 to PC3 from the principal component analysis of all complete datasets [file 13068_2022_2103_MOESM3_ESM.docx]

## Additional File 3 - Biplot of PC1 to PC3 from the principal component analysis of all complete datasets


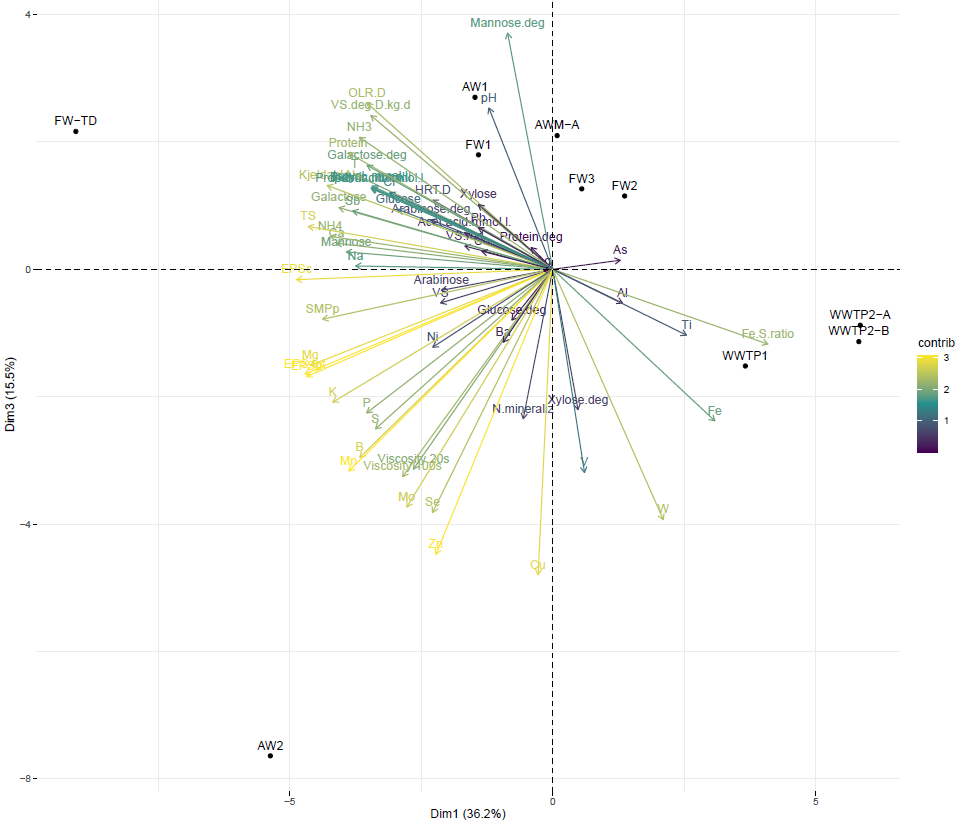


*Figure 1 Biplot of PC1 to PC3 from the principal component analysis of all complete datasets.*
