# Supplementary material for: Identifying targets for increased biogas production through chemical and organic matter characterization of digestate from full-scale biogas plants: what remains and why?
Source: Biotechnol Biofuels Bioprod. 2022 Feb 10;15:16. doi: 10.1186/s13068-022-02103-3 (PMC8830174; doi:10.1186/s13068-022-02103-3)
Supplement: Supplementary file 5 — Additional file 5. Scatter-plot for the correlation of Fe to lipase activity and to RMP [file 13068_2022_2103_MOESM5_ESM.docx]

## Additional File 5


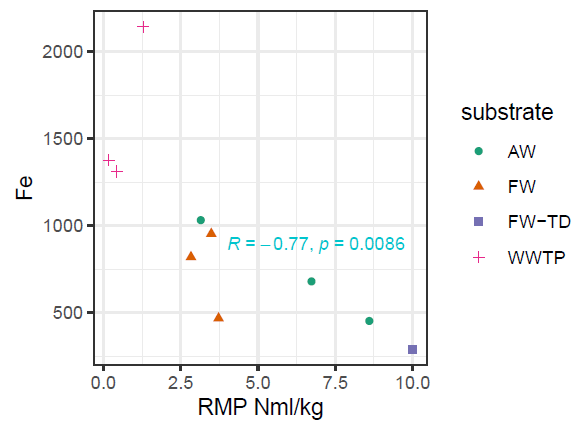

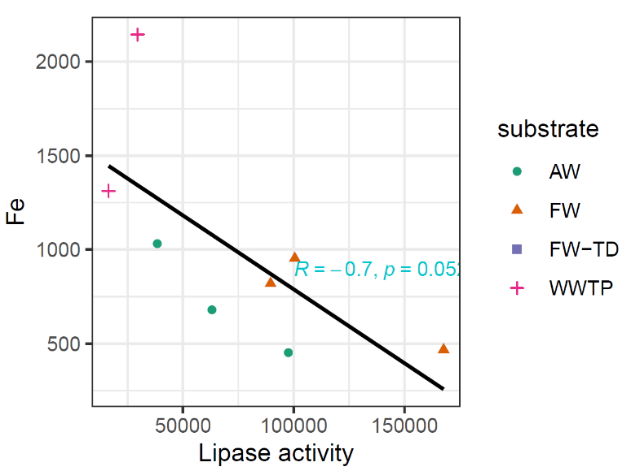


A

B

Scatter-plot for the correlation of Fe to A) lipase activity (n = 8) and B) to RMP (Nml/kg, n = 10) in the digesters of full-scale plants. AW = plant-based agricultural waste, FW = food waste, FW-TD = thermophilic dry digestion of food waste, WWTP = wastewater treatment plant
