# Supplementary material for: Identifying targets for increased biogas production through chemical and organic matter characterization of digestate from full-scale biogas plants: what remains and why?
Source: Biotechnol Biofuels Bioprod. 2022 Feb 10;15:16. doi: 10.1186/s13068-022-02103-3 (PMC8830174; doi:10.1186/s13068-022-02103-3)

## Additional file 8 – Calculation of the amount of organic material in the digested samples

The amount of organic material in the digested samples (g/kg) were adjusted to account for the reduction in volume of substrate due to gas production. The volume adjustment was based on the approximation that the reduced volume was equal to or less than the difference in total solids (TS, gram) in the volume going in (V_1_) and the volume going out (V_2_), as follows:


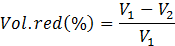


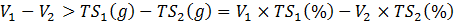


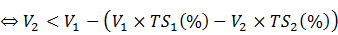


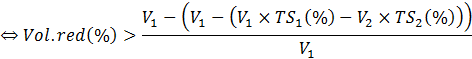


Setting V_1_ to 1:


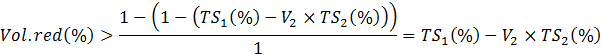


Using eq. 3 to insert into eq. 5 as an iteration to reduce the error of not knowing V_2_ (this could be done several times, the difference gets smaller every time, here only one iteration is shown before V_2_ is set to 1)


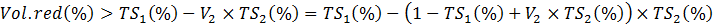


Approximating


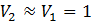


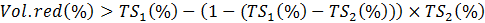


Finally, the amount of each component (i.e. protein, cellulose, lignin) was divided by the volume reduction, according to


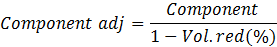

Supplement: Supplementary file 8 — Additional file 8. Calculation of the amount of organic material in the digested samples [file 13068_2022_2103_MOESM8_ESM.docx]
